# Emerging antimicrobial resistance in early and late-onset neonatal sepsis

**DOI:** 10.1186/s13756-017-0225-9

**Published:** 2017-06-13

**Authors:** Lamiaa Mohsen, Nermin Ramy, Dalia Saied, Dina Akmal, Niveen Salama, Mona M. Abdel Haleim, Hany Aly

**Affiliations:** 1 Department of Pediatrics, Faculty of Medicine, Cairo University, Cairo, Egypt; 2 Department of Clinical and Chemical Pathology, Cairo University, Cairo, Egypt; 3 Division of Neonatology, the George Washington University and Children’s National Health System, 900 23rd Street, N.W. Suite G2092, Washington, DC 20037 USA; 4 New Children Hospital, (Abu El Rish), Cairo University Hospitals, Ali Basha Ebrahim, PO Box 11562, Cairo, Egypt

**Keywords:** Blood cultures, Tracheal aspirate, *Klebsiella pneumoniae*, Gram positive cocci, Antibiotic susceptibility, Early onset sepsis, Late onset sepsis, Multidrug resistance, Neonatal sepsis

## Abstract

**Background:**

Compared to developed countries, the use of antimicrobials in Egypt is less regulated and is available over the counter without the need for prescriptions. The impact of such policy on antimicrobial resistance has not been studied. This study aimed to determine the prevalence of early and late onset sepsis, and the frequency of antimicrobial resistance in a major referral neonatal intensive care unit (NICU).

**Methods:**

The study included all neonates admitted to the NICU over a 12-month period. Prospectively collected clinical and laboratory data were retrieved, including blood cultures and endotracheal aspirate cultures if performed.

**Results:**

A total of 953 neonates were admitted, of them 314 neonates were diagnosed with sepsis; 123 with early onset sepsis (EOS) and 191 with late onset sepsis (LOS). A total of 388 blood cultures were obtained, with 166 positive results. Total endotracheal aspirate samples were 127; of them 79 were culture-positive. The most frequently isolated organisms in blood were *Klebsiella pneumoniae* (42%) and *Coagulase negative staphylococcus* (19%) whereas in endotracheal cultures were *Klebsiella pneumoniae* (41%) and *Pseudomonas aeruginosa* (19%). Gram negative organisms were most resistant to ampicillins (100%), cephalosporins (93%–100%) and piperacillin-tazobactam (99%) with less resistance to aminoglycosides (36%–52%). Gram positive isolates were least resistant to vancomycin (18%). Multidrug resistance was detected in 92 (38%) cultures, mainly among gram negative isolates (78/92).

**Conclusions:**

Antibiotic resistance constitutes a challenge to the management of neonatal sepsis in Egypt. Resistance was predominant in both early and late onset sepsis. This study supports the need to implement policies that prohibits the non-prescription community use of antibiotics.

## Background

Neonatal sepsis is a major health problem worldwide [1]. Neonates are more at risk for bacterial sepsis, with a global prevalence of 1 to 10 per 1000 live births [2]. Sepsis problem is much higher in the developing than in the developed countries, with sepsis-related mortality rate as high as 50% for untreated newborns.

Neonatal sepsis is a clinical syndrome in an infant 28 days of life or younger, manifesting with a diversity of non-specific systemic signs and symptoms and isolation of a pathogen from the bloodstream [3]. According to inception, early onset sepsis (EOS) refers to infections during the first 72 h of life that is usually related to intrapartum transmission from mothers; whereas late onset sepsis (LOS) refers to postnatal acquisition of infections after the first 3 days of life [4]. Pathogens encountered in neonatal sepsis vary worldwide; reports from developing countries more commonly show Gram negative organisms [5, 6], although Gram positive organisms have been also reported [6–8]. The susceptibility patterns for early neonatal sepsis typically differs from late neonatal sepsis; more resistant organisms are expected from hospital acquired late sepsis when compared to vertically transmitted, community acquired, early sepsis. However, a report from a developing country showed some resistant organisms to cause early neonatal sepsis [9]. Resistant organisms can potentially grow in the community with inappropriate use of antibiotics that is typical in some developing countries. Therefore, in this current study, investigators aimed to examine the microbiological patterns of early and late neonatal sepsis and to specify their antibiotic susceptibility.

## Methods

### Patients

This is a retrospective study of a prospectively collected data at the neonatal intensive care unit (NICU) of Cairo University Children’s Hospital in Egypt, conducted over a 12-month- period. The study was approved by the Ethical Committee of the hospital. Infants were included if they were diagnosed with microbiological bacteremia, and/or clinical sepsis that was accompanied by non-microbiological laboratory values suggestive of infections. Infants were considered to have sepsis if they had a score of 3 or more of the following hematologic findings: i) abnormal total leucocyte count, ii) abnormal total neutrophil (PMN) count, iii) increased immature PMN count, iv) increased immature to total PMN ratio, v) immature to mature PMN ratio ≥ 0.3, vi) platelets count ≤150,000/mm3, and vii) pronounced degenerative changes in PMNs [10]. Infants were classified into two groups according to the timing of sepsis diagnosis: EOS diagnosed ≤72 h of life and LOS diagnosed >72 h of life. Demographic, clinical, and laboratory data were retrieved for all included infants.

### Infectious control management

All admitted infants received a limited sepsis work up that included complete blood count, C-reactive protein, and blood culture. Empiric parenteral antibiotics were initiated for 3 days while awaiting blood culture results. Sepsis work ups were repeated during hospital stay whenever an infant displayed clinical signs suggestive for sepsis. Infants were considered to have bloodstream infection if they had at least one blood culture positive for organisms known to cause bacteremia [11]. For other organisms that may cause true bacteremia or may represent skin contamination, infection was considered if the same organism was recorded from at least two blood cultures. Endotracheal aspirate (ETA) cultures were occasionally obtained from mechanically ventilated cases if they displayed clinical signs suggestive of ventilator associated pneumonia such as increased oxygen requirement, increased ventilator support settings, worsening radiographic findings, and changes in tracheal aspirate volume, color or consistency.

### Microbiological sampling

For blood cultures, at least 1 ml of blood sample was obtained from a peripheral vein under aseptic precautions. Blood samples were incubated in blood culture incubator (BACTEC- 9050, Beckton-Dickenson, Franklin Lakes, New Jersey, USA). ETA samples were obtained by direct endotracheal suction of respiratory secretions using sterile endotracheal suction catheters into sterile suction trap.

### Sample processing

Specimens were processed on arrival to the laboratory. Enriched media used included: blood, MacConkey and chocolate agar plates. These were inoculated, incubated at 37 °C, and examined for growth at 24–48 h. Isolates, if any, were identified by: Gram staining, colony characteristics, and biochemical properties including catalase, DNAse agar, mannitol salt agar, and hemolysis on blood agar plates, for Gram positive isolates, and triple sugar iron (TSI), lysine iron agar (LIA), motility indole, ornithine (MIO), citrate, urease and oxidase for Gram negative bacilli [12]. All media and biochemical reactions were systematically quality controlled according to the standards by the American Type Culture Collection. Antibiotic susceptibility tests were performed by Kerby-Bauer disc diffusion method according to the standards of Clinical and Laboratory Standards Institute (CLSI). The antibiotic discs used represented different groups of antibiotics. The inhibition zones were measured and interpreted according to the CLSI recommendations [13]. Multi drug resistant (MDR) bacteria were defined by resistance to three or more antimicrobial classes [14].

### Statistical analysis

All statistical procedures were performed using the Statistical Package for Social Science (SPSS) for windows version 16.0 (SPSS Inc., Chicago, Illinois, USA). Descriptive analyses were expressed as mean ± standard deviation (SD) for quantitative variables, and percentages (%) for categorical variables. Differences in distribution for categorical variables were done using the Chi square test.

## Results

Nine hundred fifty three cases were admitted during the study period; of them 314 (32.9%) neonates were diagnosed with sepsis based on clinical signs and/or microbiological laboratory. Early onset sepsis was detected in 123 cases and late onset sepsis in 191 cases. The characteristics of the study population are presented in Table 1. Seventy-seven cases were admitted solely for sepsis whereas 237 had other associated morbidities as shown in Table 2. Sepsis presented more frequently in males than females (178 vs. 136).

**Table 1:** Demographic characteristics of the study population

Variable	Early onset sepsis (*n* = 123)	Late onset sepsis (*n* = 191)
- Gestational age (weeks)		
≤ 28 weeks (n) (%)	10 (8.13%)	12 (6.28%)
> 28–32 weeks (n) (%)	40 (32.52%)	21 (10.99%)
> 32–36 weeks (n) (%)	32 (26.01%)	43 (22.51%)
> 36 weeks (n) (%)	41 (33.33%)	115 (60.20%)
- Male/Female (n)	72/51	106/85
- Age on admission (days) (mean ± SD)	2.41 ± 4.1	9.96 ± 9.2
- Length of hospital stay(days) (mean ± SD)	16.46 ± 16.48	18.68 ± 16.61
- Outcome (deaths) (n) (%)	38 (30.8%)	32 (16.8%)

**Table 2:** Clinical presentations and diagnoses among studied cases with suspected sepsis

Clinical manifestation/ diagnosis	Number (%)
- Poor suckling & sluggish reflexes	77 (24.5%)
- Respiratory distress	91 (29.0%)
- Pulmonary hypertension	3 (1.0%)
- Meconium aspiration syndrome	4 (1.3%)
- Apnea	35 (11.2%)
- Congenital heart disease	6 (1.9%)
- Neonatal jaundice	19 (6.1%)
- Neonatal convulsions	12 (3.8%)
- Hypoxic ischemic encephalopathy	11 (3.5%)
- Neonatal hypoglycemia	7 (2.2%)
- Multiple congenital anomalies	10 (3.2%)
- Low birth weight	10 (3.2%)
- Neonatal Pneumonia	15 (4.8%)
- Bleeding tendency or hemorrhage	14 (4.5%)
- Total	314(100%)

A total of 388 blood specimens were cultured from our 314 septic neonates, with more than one culture obtained from the same case in some occasions, with 166 microbiologically positive cultures; of them 49 cultures for cases with EOS and 117 cultures for cases with LOS. The most common organisms for EOS and LOS were gram negative bacilli (31/49 and 87/117 respectively), mainly *Klebsiella pneumoniae spp. (n =* 20 for EOS, and 49 for LOS). Table 3 represents the isolated organisms in the studied population.

**Table 3:** Organisms in blood cultures of infants with early and late onset sepsis (*n* = 166)

	Blood culture		
Isolated organism	Early onset sepsis	Late onset sepsis	Total
-*Klebsiella pneumoniae*	20	49	69 (41.56%)
*-Pseudomonas aeruginosa*	7	14	21 (12.65%)
*-Acinetobacter*	3	10	13 (7.83%)
*-CONS*	15	16	31 (18.67%)
*-MRSA*	2	7	9 (5.42%)
*-B streptococci*	0	4	4 (2.40%)
*-Streptococcus pneumoniae*	1	0	1 (0.60%)
*-Staphylococcus aureus*	0	3	3 (1.80%)
*-Escherichia coli*	0	4	4 (2.40%)
*-Enterobacter*	1	7	8 (4.81%)
*-Salmonella*	0	1	1 (0.60%)
*-Stenotrophomonaes*	0	2	2 (1.20%)
-Total			166

*CONS* coagulase negative staphylococci, *MRSA* methicillin resistant staphylococcus aureus

A total of 127 endotracheal aspirates (ETA) were obtained during the study period from 58 patients with 79 positive cultures; of them 24 cultures belonged to cases with EOS and 55 with LOS. The most prominent organisms in ETA cultures were *Klebsiella pneumoniae* in cases with EOS (22/24) and *Pseudomonas aeruginosa* (21/55) in LOS cases (Table 4). Organisms isolated from ETA cultures differed from those in blood cultures in 29% of cases.

**Table 4:** Organisms isolated in endotracheal aspirate cultures of infants (*n* = 79)

	ETA culture
Isolated organism	Total
-*Klebsiella Pneumoniae*	36 (45.6%)
*-Pseudomonas aeruginosa*	23 (29.1%)
*-Acinetobacter*	11 (13.9%)
*-Escherichia coli*	2 (2.5%)
*-Enterobacter*	4 (5.1%)
*-CONS*	2 (2.5%)
*-Staphylococcus aureus*	1 (1.3%)
Total	79

*CONS* coagulase negative staphylococci

Antimicrobial sensitivity and resistance patterns were assessed for all 245 isolated bacteria; 166 blood cultures and 79 ETA cultures. Tables 5 and 6 present the susceptibility patterns of gram negative bacilli and gram positive cocci respectively. Gram negative bacilli showed highest resistance to ampicillins (ampicillin- sulbactam, 100% and amoxicillin-clavulanate, 97%), cephalosporins (cefotaxime, 93%, ceftazidime, 96%, cefoperazone, 95%, ceftriaxone, 99%, cefuroxime, 100%), and piperacillin-tazobactam, 99%. Less resistance was evident to aminoglycosides, mainly amikacin (36%) and gentamicin (52%), as well as carbapenems (imipenem, 26% and meropenem, 64%). Least resistance to quinolones (levofloxacin, 24%). Gram positive cocci showed highest resistance to ampicillins (amoxicillin-sulbactam, 100% and amoxicillin-clavulanate, 75%), cephalosporins (ceftazidime, 94%, cefoperazone, 100%, cefepime, 86%, ceftriaxone, 100%, cefuroxime, 100%, cefoxitin, 80%), carbapenems (imipenem, 84%, meropenem, 86%), piperacillin-tazobactam (100%), and erythromycin (86%). Less resistance was evident to aminoglycosides (amikacin, 49%, gentamicin, 57%), quinolones (ciprofloxacin, 77%, levofloxacin, 75%), clindamycin (53%), and rifampicin (49%). Least resistance among gram positive bacteria was found to vancomycin (18%). Multidrug resistance was detected in 67 cases; 18 with EOS and 49 with LOS, and in 92 (37.6%) cultures, mainly among gram negative isolates (78/92).

**Table 5:** Resistance of gram negative bacilli to various antimicrobials

Organism (number)	Ampicillin-sulbactam	Amikacin	Cefotaxime	Ceftazidime	Imipenem	Vancomycin	Ciprofloxacin	Clindamycin		Rifampicin	Piperacillin-tazobactam
*Kl(105)*	105(100%)	45(43%)	103(98.1%)	105(100%)	22(21%)	ND	48(45.7%)	ND		ND	103(98.1%)
*Pseudomonas (44)*	44(100%)	12(27.3%)	39(88.6%)	41(93.2%)	17(38.6%)	ND	22(50%)	ND		ND	44(100%)
*Acinetobacter (24)*	24(100%)	8(33.3%)	20(83.3%)	21(87.5%)	9(37.5%)	ND	8(33.3%)	ND		ND	24(100%)
*Salmonella (1)*	1(100%)	1(100%)	1(100%)	1(100%)	0	ND	0	ND		ND	1(100%)
*E coli (6)*	6(100%)	1(16.7%)	4(66.7%)	5(83.3%)	0	ND	2(33.3%)	ND		ND	6(100%)
*Enterobacter (12)*	12(100%)	3(25%)	12(100%)	12(100%)	2(16.7%)	ND	5(41.7%)	ND		ND	12(100%)
*Stenotrophomonas(2)*	2(100%)	0	2(100%)	2(100%)	1(50%)	ND	2(100%)	ND		ND	2(100%)
	Gentamicin	Meropenem	Cefoperazone	Cefepime	Amoxicillin-clavulanate	Ceftriaxone	Cotrimoxazole	Erythromycin	Cefoxitin	Levofloxacin	Cefuroxime
*Kl (105)*	63(60%)	34(32.4%)	103(98.1%)	86(81.9%)	101(96.2%)	103(98.1%)	86(81.9%)	ND	82(78.1%)	25(24%)	105(100%)
*Pseudomonas (44)*	21(47.7%)	20(45.5%)	39(88.6%)	28(63.6%)	43(97.7%)	44(100%)	37(84.1%)	ND	38(86.4%)	8(18%)	44(100%)
*Acinetobacter (24)*	7(29.2%)	10(41.7%)	21(87.5%)	14(58.3%)	24(100%)	24(100%)	19(79.2%)	ND	16(66.7%)	7(29%)	24(100%)
*Salmonella (1)*	1(100%)	0	1(100%)	1(100%)	1(100%)	1(100%)	0	ND	1(100%)	0	1(100%)
*E coli (6)*	1(16.7%)	0	6(100%)	5(83.3%)	6(100%)	6(100%)	2(33.3%)	ND	2(33.3%)	1(17%)	6(100%)
*Enterobacter(12)*	8(66.7%)	4(33.3%)	12(100%)	8(66.7%)	11(91.7%)	12(100%)	10(83.3%)	ND	11(91.7%)	3(25%)	12(100%)
*Stenotrophomonas(2)*	0	1(50%)	2(100%)	2(100%)	2(100%)	2(100%)	2(100%)	ND	1(50%)	2(100%)	2(100%)

*ND* Sensitivity was not done, *E coli Escherichia coli, Klebsiella Klebsiella pneumoniae, Pseudomonas Pseudomonaas aeruginosa*

**Table 6:** Resistance of gram positive cocci to various antimicrobials

Organism (number)	Ampicillin-sulbactam	Amikacin	Cefotaxime	Ceftazidime	Imipenem	Vancomycin	Ciprofloxacin	Clindamycin	Polymyxin	Rifampicin	Piperacillin-tazobactam
*CONS (33)*	33(100%)	16(48.5%)	31(93.9%)	31(93.9%)	28(84.8%)	8(24.2%)	25(75.8%)	18(55%)	ND	15(46%)	33(100%)
*MRSA (9)*	9(100%)	3(33.3%)	9(100%)	9(100%)	8(88.9%)	0	8(88.9%)	3(33%)	ND	3(33%)	9(100%)
*B streptococcus (4)*	4(100%)	2(50%)	3(75%)	3(75%)	2(50%)	1(25%)	3(75%)	3(75%)	ND	4(100%)	4(100%)
*St. pneumonie (1)*	1(100%)	1(100%)	1(100%)	1(100%)	1(100%)	0	0	1(100%)	ND	1(100%)	1(100%)
*Staph aureus (4)*	4(100%)	3(75%)	4(100%)	4(100%)	4(100%)	0	3(75%)	2(50%)	ND	2(50%)	4(100%)
	Gentamicin	Meropenem	Cefoperazone	Cefepime	Amoxicillin-clavulanate	Ceftriaxone	Cotrimoxazole	Erythromycin	Cefoxitin	Levofloxacin	Cefuroxime
*CONS (33)*	18(55%)	28(85%)	33(100%)	29(88%)	26(79%)	33(100%)	27(82%)	28(85%)	25(76%)	22(67%)	33(100%)
*MRSA (9)*	7(77.8%)	9(100%)	9(100%)	9(100%)	9(100%)	9(100%)	8(89%)	7(78%)	9(100%)	7(78%)	9(100%)
*B streptococcus (4)*	0	2(50%)	4(100%)	2(50%)	3(75%)	4(100%)	4(100%)	4(100%)	3(75%)	4(100%)	4(100%)
*St. pneumonie (1)*	0	1(100%)	1(100%)	0	0	1(100%)	1(100%)	1(100%)	1(100%)	1(100%)	1(100%)
*Staph aureus (4)*	4(100%)	4(100%)	4(100%)	4(100%)	0	4(100%)	4(100%)	4(100%)	3(75%)	4(100%)	4(100%)

*ND* sensitivity was not done, *CONS coagulase negative staphylococci, MRSA methicillin resistant staphylococcus aureus, St streptococcus, Staph. aureus Staphylococcus aureus*

## Discussion

Neonatal sepsis, a life-threatening condition, needs immediate empirical antimicrobial therapy. It is important to choose an antibiotic combination that covers the most common pathogens [15]. Blood culture remains the gold standard for diagnosis of neonatal sepsis, despite its low sensitivity which may be due to small volume of blood sample, or empirical antibiotics prior to sampling [16].

We observed the emergence of multi drug resistance among cases of EOS, which has not been frequently mentioned. A previous study from India stated that multidrug resistant organisms were leading causes of early as well as late onset sepsis [17]. Even studies from Egypt addressing this problem studied multi drug resistance either collectively [18] or in relation to late onset sepsis only [19]. The worrisome rise in levels of antimicrobial resistance among pathogens retrieved from NICUs highlight the needs for better understanding of the problem of early onset sepsis and implementing strategies to combat, especially in countries with limited resources [20].

In the current study, the overall incidence of suspected sepsis was 32.9%. These results are comparable to other studies from Egypt [21, 22], but are better than a previous report in 2001 wherein the rate of sepsis exceeded 50% [23], which may be due to better awareness and adherence to infection control measures.

In this study, the overall mortality in septic neonates was 22.3%. This value is lower than sepsis related deaths reported in other studies from Egypt [21, 23], however, several studies reviewed from different developing countries showed a wide range of infection related neonatal mortality ranging between 8 and 80% [24]. A sepsis related mortality of 19% was reported in studies from east Africa [25, 26].

In this work, the incidence of LOS was higher than that of EOS. Comparable findings were reported in other studies from Egypt and South Africa [21, 27]. The opposite was found in studies from Nepal [28] and Iran [29].

Suspected sepsis was confirmed by blood culture, yielding different bacterial growths in only 166/388 cultures (42.8%). This rate is in the vicinity to those of other studies from Egypt [21] and other developing African and Asian countries [25, 30]. Despite being the gold standard for diagnosing sepsis, blood cultures suffer from low sensitivity, postulated reasons include prior use of antibiotics, insufficient or wrong sampling, poor transport conditions, and slow-growing or fastidious bacteria [31]. Also, some culture negative patients might be due to non-bacterial causes as fungi, viruses and parasites [32]. ETA cultures yielded matching results to blood cultures in 38% of simultaneous cultures and grew different organisms in 29% of cases. Authors showed ETA cultures to be of no value in predicting pathogens causing septicemia in ventilated infants [33]. Higher percentages of matching cultures in this study may be explained by higher simultaneous negative cultures.

Gram negative bacilli were more frequently encountered than gram positive cocci, with *Klebsiella pneumoniae* being the most commonly isolated organism both in blood and ETA cultures (42% and 41% respectively),. These results were consistent in multiple reports from Egypt over two decades [22, 34, 35]. However, other reports from Egypt showed *CONS* as the leading cause of sepsis in 2006 [36], 2010/2011 [37], and 2011/2012 [21]. These results support the fact that the diversity of organisms causing sepsis varies from region to another and changes over time even in the same place [38].

The frequency of gram negative pathogens varied from 31% to 63% with *Klebsiella pneumoniae, Pseudomonas aeruginosa* and *Escherichia coli* being the predominant organisms in almost all countries of Latin America [39]. Several other studies in a diversity of developing countries showed that gram negative bacteria were responsible for most cases of neonatal sepsis [29, 30, 40, 41]. Although some authors state that gram-positive bacteria are the most commonly encountered in NICU patients [42, 43], yet case fatalities are highest for gram-negatives [44]. Others have indicated increasing incidence of gram-negative bacterial infections in NICUs [45].

Among gram negative organisms, *Klebsiella pneumoniae* is increasingly emerging as a common bacteria in hospital settings [46].

The first line of empirical treatment in this NICU is Ampicillin-sulbactam combined with cephalosporins or aminoglycosides. In the absence of clinical improvement, antibiotics are changed to carbapenems and vancomycin until the blood culture results are available. Quinolones are used in culture-proven sepsis with multidrug resistant organisms. In this study, gram negative organisms were most resistant to ampicillins, cephalosporins, and piperacillin- tazobactam. Less resistance was observed to aminoglycosides and carbapenems with least resistance to levofloxacin. Several studies showed high resistance to ampicillin and amoxicillin, aminoglycosides, and different classes of cephalosporins [41, 47]. Even within the aminoglycoside spectrum, some authors found amikacin (which was less used in their units) more sensitive than gentamicin (which was more commonly used) [47]. Two studies in sub-Saharan Africa and Asia revealed resistance of the two common pathogens *Klebsiella* and *Staphylococcus aureus* to almost all commonly used antibiotics in one study [48], and *Klebsiella pneumoniae* median resistance to ampicillins and cephalosporins in 94 and 84% of cases in Asia and 100 and 50% in Africa [49] in the other study. Although short courses of antimicrobials as carbapenems and cephalosporins especially third generation cover a broad spectrum of bacteria, yet their extended use caused the emergence of extended spectrum β lactamase producing gram negative bacteria. This confers resistance to penicillins and cephalosporins and often coexisting with resistance to other categories of antibiotics as quinolones and aminoglycosides [50–53]. In Egypt, gram negative bacteria were resistant to ampicillin, amoxicillin clavulanate and cephalosporins, with highest sensitivity to either or both carbapenems and quinolones [21, 35, 37].

The increasing multidrug resistant gram negative bacteria with relative deficiency of new antibiotics to combat led to the revival of other classes of drugs as polymyxins, which are active against *Acinetobacter* species, *Pseudomonas aeruginosa*, *Klebsiella* species, and *Enterobacter* species [54].

In this work, gram positive organisms were most resistant to ampicillins (mainly ampicillin-sulbactam), a wide variety of cephalosporins (ceftazidime, cefoperazone, cefepime, ceftriaxone, cefoxitime, cefuroxime), carbapenems, piperacillin-tazobactam, and erythromycin. They were less resistant to aminoglycosides, quinolones, clindamycin, and rifampicin and least resistant to vancomycin.

In one study in India, rifampicin was effective against *Staphylococcus aureus* [55]. Other drugs as erythromycin show rising resistance among streptococci species (mainly *group B streptococci, group A streptococci, Streptococcus pyogenes*, and *Streptococcus pneumoniae*) and *Staphylococcus aureus* [56]. Similarly, in current work, erythromycin resistance was highest against *Streptococcus pneumoniae, group B streptococci*. There is an increasing incidence of multi drug resistant gram positive organisms [57], as well as increasing vancomycin resistant isolates [58], however; vancomycin still is an important first-line antimicrobial for treatment of serious infections as *Methicillin-Resistant Staphylococcus aureus* [59]. In our study, we found high resistance to fluoroquinolones among gram positive organisms. This resistance is emerging due to their extensive and increasing use in medical practice and was stated in the literature to be greatest in *Staphylococcus aureus* isolates especially methicillin resistant strains [60].

## Conclusion

This study demonstrated a high prevalence of gram negative bacilli sepsis and tracheal colonization. Both gram negative bacilli and gram positive cocci were highly resistant to multiple broad-spectrum antimicrobials. Resistance was the highest against antibiotics used frequently in the NICU as the first line or the second line empirical treatment. It is worrisome to discover that pan drug resistance was not restricted to LOS; infants admitted from the community with early onset sepsis had a high resistance index to the multiple antibiotics. Of note, antibiotics in Egypt are available over the counter and do not require a physician’s prescription. This study calls for global regulations to restrict the use of antimicrobials in the community as well as in the hospital setting.

## Abbreviations

CLSI: Clinical and Laboratory Standards Institute
EOS: Early onset sepsis
ETA: Endotracheal aspirate
LIA: Lysine iron agar
LOS: Late onset sepsis
MDR: Multi drug resistance
MIO: Motility indole ornithine
NICU: Neonatal ntensive Care Unit
PMN: Polymorph nuclear leucocytes
spp.: Species
SPSS: Statistical Package for Social Science
TSI: Triple sugar iron
